# Left Atrial Appendage Volume Increased in More Than Half of Patients with Cryptogenic Stroke

**DOI:** 10.1371/journal.pone.0079519

**Published:** 2013-11-04

**Authors:** Mikko Taina, Ritva Vanninen, Marja Hedman, Pekka Jäkälä, Satu Kärkkäinen, Tero Tapiola, Petri Sipola

**Affiliations:** 1 Department of Clinical Radiology, Kuopio University Hospital, Kuopio, Finland; 2 Unit of Radiology, Institute of Clinical Medicine, University of Eastern Finland, Kuopio, Finland; 3 Heart Center, Kuopio University Hospital, Kuopio, Finland; 4 NeuroCenter, Kuopio University Hospital, Kuopio, Finland; 5 Unit of Neurology, Institute of Clinical Medicine, University of Eastern Finland, Kuopio, Finland; 6 Department of Neurology, North Kymi Hospital, Kouvola, Finland; The University of Chicago, United States of America

## Abstract

**Background:**

Ischemic strokes without a well-defined etiology are labeled as cryptogenic, and account for 30–40% of strokes in stroke registries. The left atrial appendage (LAA) is the most typical origin for intracardiac thrombus formation when associated with atrial fibrillation. Here, we examined whether increased LAA volume detected with cardiac computed tomography (cCT) constitutes a risk factor in cryptogenic stroke patients.

**Methods:**

This study included 82 stroke/TIA patients (57 males; mean age, 58 years) with a diagnosis of cryptogenic stroke after extensive radiological and cardiological investigations. Cases were classified using the TOAST criteria modified by European Association of Echocardiography recommendations for defining cardiac sources of embolism. Forty age- and gender-matched control subjects without cardiovascular diseases were selected for pair-wise comparisons (21 males; mean age, 54 years). LAA volume adjusted for body surface area was measured three dimensionally by tracing the LAA borders on electrocardiogram-gated CT slices.

**Results:**

In control subjects, mean LAA volume was 3.4±1.1 mL/m^2^. Mean+2SD, which was considered the upper limit for normal LAA volume was 5.6 mL/m^2^. In paired Student t-test between the patient group and matched controls, LAA volume was 67% larger in cryptogenic stroke/TIA patients (5.7±2.0 mL/m^2^ vs. 3.4±1.1 mL/m^2^; *P*<0.001). Forty-five (55%) patients with cryptogenic stroke/TIA had enlarged LAA.

**Conclusion:**

LAA is significantly enlarged in more than half of patients with cryptogenic stroke/TIA. LAA thrombosis may contribute to the pathogenesis of stroke in patients considered to have cryptogenic stroke after conventional evaluation.

## Introduction

Stroke is the leading cause of long-term disability, and a major consumer of health-care resources worldwide [1]. It causes 10% of all deaths and is the second highest cause of mortality [1,2].The currently recognized ischemic stroke mechanisms are embolism, decreased perfusion, and thrombosis [3]. Embolism to the brain can be arterial or cardiac in origin, with atrial fibrillation (AF) being by far the most common cause of cardioembolic stroke [4,5]. Ischemic strokes without a well-defined etiology are labeled as cryptogenic, and account for 30–40% of all ischemic strokes [6]. It has been suggested that cardiac embolism may also constitute a major mechanism for cryptogenic stroke [7,8]. In a recent multicenter study, outpatient cardiac telemetry over 21 days detected occult paroxysmal atrial fibrillation (PAF) in almost 20% of patients with cryptogenic cerebral ischemia [9].

Most cardiac thrombi originate from the left atrial appendage (LAA) [10]. An enlarged LAA may predispose to blood coagulation due to slow flow velocity [11]. MRI results have shown enlarged LAA in stroke/TIA patients with AF [12–14]. Moreover, enlarged left atrium (LA) and LAA could indicate a propensity for PAF, regardless of sinus rhythm at the time of ischemic episode [15]. The present study aimed to assess whether LAA and/or LA volume is increased in patients with cryptogenic stroke/TIA.

## Methods

The study was approved by the University Hospital Research Ethics Board. Prior to participation in the study, written informed consent was obtained from the patient or the patient's legally authorized representative.

### Study Design and Population

**Figure 1:**
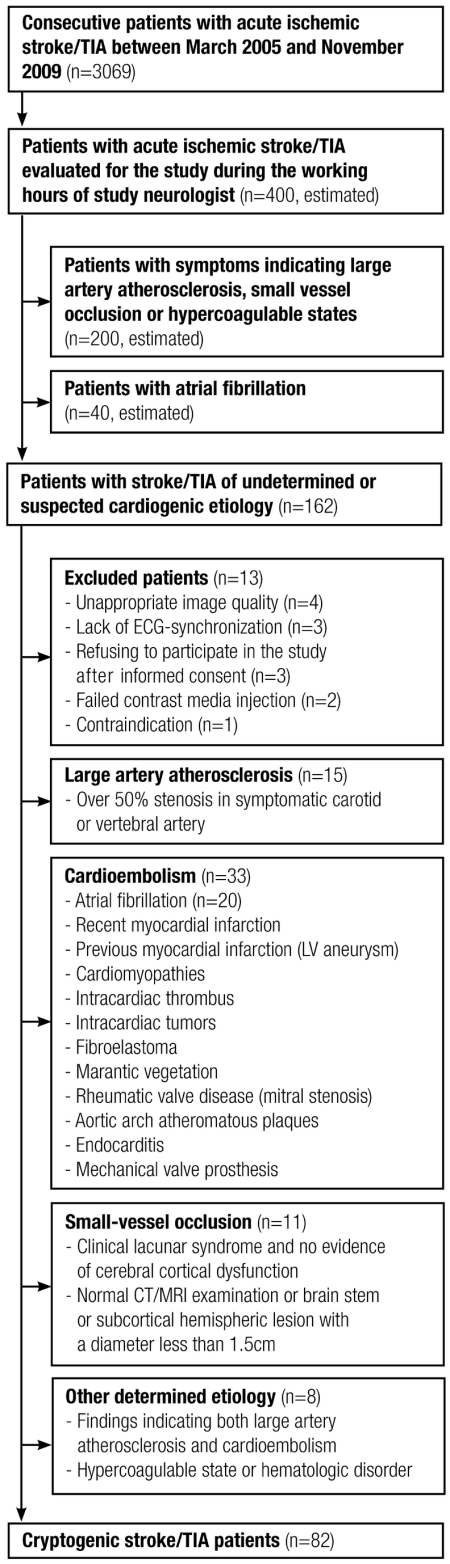
Flow chart of patient recruitment. Neurologists recruited consecutive patients with acute ischemic stroke/TIA with undetermined etiology or a suspicion of cardiogenic etiology. Stroke/TIA patients with atrial fibrillation were excluded. Thirteen patients were excluded from the study after recruitment. The remaining patients were subjected to thorough clinical, cardiological and radiological examinations. Patients were further categorized according to the TOAST classification, denoting five subtypes of ischemic stroke: 1) large-artery atherosclerosis, 2) cardioembolism, 3) small-vessel occlusion, 4) stroke of other determined etiology, and 5) stroke of undetermined etiology. The classification was updated by applying the more recent EAE recommendations for defining cardiac sources of embolism.

Patients with acute stroke/TIA who were admitted to our university hospital were evaluated as candidates for this cardiac CT (cCT) study, EMBODETECT (Figure 1) [16]. The neurologists involved in this study recruited 162 patients with stroke/TIA of undetermined etiology or with suspected cardioembolic etiology other than atrial fibrillation, based on the characteristic clinical symptoms and/or primary clinical signs, i.e., simultaneous or sequential strokes/TIAs in different arterial territories, hemorrhagic transformation, simultaneous emboli in other organs, decreased consciousness at stroke/TIA onset, isolated aphasia, or isolated visual-field defect. Patients with AF recorded in their patient file or during time of enrolment were excluded.

Of the 162 patients initially recruited, 13 were excluded; the cCT image quality was not appropriate for LAA size analyses in four patients, ECG-synchronization was not used for cardiac imaging in three patients, contrast media injection failed in two patients, use of contrast media was contraindicated due to increased serum creatinine level in one patient, and three patients refused to participate after giving informed consent.

Transthoracic (TTE) and transesophageal echocardiography (TEE) were performed with the Vivid 7 cardiovascular ultrasound system (M4S 3 MHz probe for transthoracic and 6T multiplane probe for transesophageal echocardiography; GE Medical Systems, Buckinghamshire, UK), by several cardiologists as part of their clinical routine. Contrast-enhanced cardiac MRI (0.2 mmol/kg Dotarem; Guerbet, Paris, France) was performed using a 1.5T scanner and 12-element phased-array surface coil (Siemens Avanto, Erlangen, Germany), to confirm suspected structural abnormalities in cCT (n=18) or in patients with discrepant findings in cCT and echocardiography (n=12). Imaging sequences and orientations were planned according to the protocols of the Society of Cardiovascular Magnetic Resonance for left ventricle (LV) structure, function, and late gadolinium enhancement modules. Ambulatory 24-hour Holter ECG was performed to exclude PAF.

After profound investigations, patients with a defined etiology for stroke (n=67) were further excluded. Large-artery atherosclerosis was found in 15 patients (10%); cardioembolism in 33 patients (22%), including 20 patients (12% of the 162 study patients) having PAF in a 24-hour Holter ambulatory ECG recording; small-vessel occlusion in 11 patients (7%); and stroke of other determined etiology in 8 patients (5%). We modified the TOAST classification for cardioembolism, applying the more recent European Association of Echocardiography (EAE) recommendations for defining cardiac sources of embolism [17,18]. The remaining 82 patients (57 males; mean age 58±10 years, range 32–82) were considered to have had an acute stroke/TIA of cryptogenic etiology.

### Control subjects

Between December 2008 and July 2011, 243 consecutive patients underwent coronary CT-angiography (coronary CTA) for exclusion of coronary artery disease. We excluded subjects with coronary stenosis of ≥50%, AF, hypertension, renal insufficiency, malignancies, or any neurological diagnosis including any symptoms indicating stroke or TIA —leaving 124 suitable candidates for pair-wise comparisons. No additional imaging such as head CT, cervicocranial CT angiography or MRI were performed for control subjects. Forty of these patients (21 males; mean age 54±9 years, range 32–74) were used as controls to create age- and gender-matched pairs with patients with cryptogenic stroke/TIA.

### Assessment of LAA and LA Size

#### CT imaging

Contrast-enhanced cCT was performed with a 16- (113 patients) or 64-slice (36 patients, 40 control subjects) scanner (Somatom Sensation 16 and Somatom Definition AS; Siemens Medical Solutions, Forchheim, Germany). Control subjects with initial heart rates higher than 65 beats per minute received 5–20 mg metoprolol intravenously before examination. In stroke patients, the aortic arch and cervical and intracranial arteries were scanned first, immediately followed by scanning of the ascending aorta and heart. When using the 16-slice scanner, contrast agent was injected through an 18-gauge catheter into the antecubital vein at 5 mL/s, followed by a 50-mL injection at 2 mL/s and a subsequent 20-mL saline chaser. With the 64-slice scanner, 100 mL of contrast agent (350 mgI/mL) was injected at 5 mL/s, followed by a 20-mL injection at 2 mL/s and a subsequent 20-mL saline chaser. For coronary CTA, 80 mL of contrast agent (350 mgI/mL) was injected at 5 mL/s, followed by an 80-mL saline chaser at 5 mL/s. Cardiac imaging was performed during mid-diastole in all study subjects. In the 16-slice scanner, collimation was 16×0.75 mm, rotation time was 0.42 s, and tube potential was 120 kV; the current was set to 500 mAs for the first 80 patients and reduced to 250 mAs thereafter. In the 64-slice scanner, collimation was 64×0.6 mm, rotation time 0.33 s, and tube potential 120 kV; the reference current was set using commercially available tube current modulation software (CAREDose4D, Siemens Medical Solutions), at 160 mAs for the first 15 patients and then reduced to 100 mAs. In coronary CTA, the corresponding values were 64×0.75 mm, 0.17 s, 120 kV, and 327 mAs, respectively. We calculated the product of CTDIvol and scanning length (dose-length product in milligrays times centimeters), and estimated the effective dose (in millisieverts) using a normalization factor for the adult chest (0.017 mSv·mGy^−1^·cm^−1^). The radiation dose per patient was 10.0±3.5 mSv in patients with stroke and 7.9±2.3 mSv in patients with coronary CTA. Mid-diastolic 0.75- to 1.0-mm-thick slices with 20–25% overlap were reconstructed. We also reconstructed 3-mm-thick transversal slices without gap or overlap for quantitative measurements.

#### Volumetric analysis of LAA and LA

Quantitative image analysis was performed on an IDS5 diagnostic workstation (version 10.2P4; Sectra Imtec, Linköping, Sweden) using magnified images on 1024×768 and 1600×1200 displays. Due to contrast agent injection, the LAA was seen as a hyperdense structure surrounded by hypodense pericardial fat, with high tissue contrast between the anatomical structures. The entire LAA was fully opacified with contrast media in all study subjects. A two-chamber view and localizer tool were used to differentiate the LAA orifice from the LA. The LAA borders were traced manually on the transversal slices. LAA trabeculations were considered part of the LAA cavity. LA borders were traced from the two-chamber plane using the mitral valve annulus as the landmark differentiating the LA from the LV. Planimetration of the LAA totally covered 10.4±2.0 slices in the transversal plane, and planimetration of the LA covered 20.0±3.2 slices in the two-chamber plane. LAA volume was calculated with Simpson’s method by multiplying each manually traced LAA and LA area by the section thickness (3 mm) and summing up the volumes of the separate sections [19]. Volumes of LAA and LA are presented as unadjusted, and adjusted for height and body surface area (BSA). To define BSA-adjusted LAA volume, BSA was calculated using Mosteller’s formula [20]. Image analysis was performed by an independent observer (M.T.), guided by a radiologist with 15 years of experience with cardiac imaging (P.S.). To calculate intraobserver variability, Observer 1 (blind to previous measurements) reconstructed new slices and repeated the LAA and LA measurements of 40 patients one month later. To calculate interobserver variability, Observer 2 reconstructed new slices and analyzed LAA and LA volumes of the same 40 patients.

### Statistical Analyses

Continuous variables with normal distribution are presented as mean±SD, and categorical variables as absolute values and percentages. An enlarged LAA was defined as one with a volume exceeding the mean volume in the control population by at least two SD. Based on normal distribution in Kolmogorov-Smirnov test, Student’s *t*-test was used to compare LAA volumes between stroke/TIA patients and control subjects. Analysis of covariance (ANCOVA) was used to control for the effects of BMI and hypertension when comparing the LAA and LA sizes between stroke/TIA patients and control subjects. Pearson’s correlation coefficient was used to investigate the associations between continuous variables, and chi-square test to investigate nominal variables. Statistical significance was set at *P*<0.05 and high statistical significance at *P*<0.01. The significance of differences in the proportions of enlarged LAAs and enlarged LAs among stroke/TIA patients was tested using the McNemar test of proportions. Cohen’s kappa coefficient was used to measure dependence of the LAA enlargement on the LA enlargement; kappa values >0.75 were categorized as high dependence, 0.40–0.75 as moderate dependence, and <0.40 as minor dependence. Intraclass correlation of coefficients (ICC) and coefficients of variabilities (CV%) were calculated to assess the reproducibility of LAA and LA measurements. Data were analyzed using SPSS for Windows (version 19, 1989–2010 SPSS Inc., Chicago, USA).

## Results

### LAA and LA Volume in Matched Patients with Cryptogenic Stroke/TIA and Control Subjects

**Figure 2:**
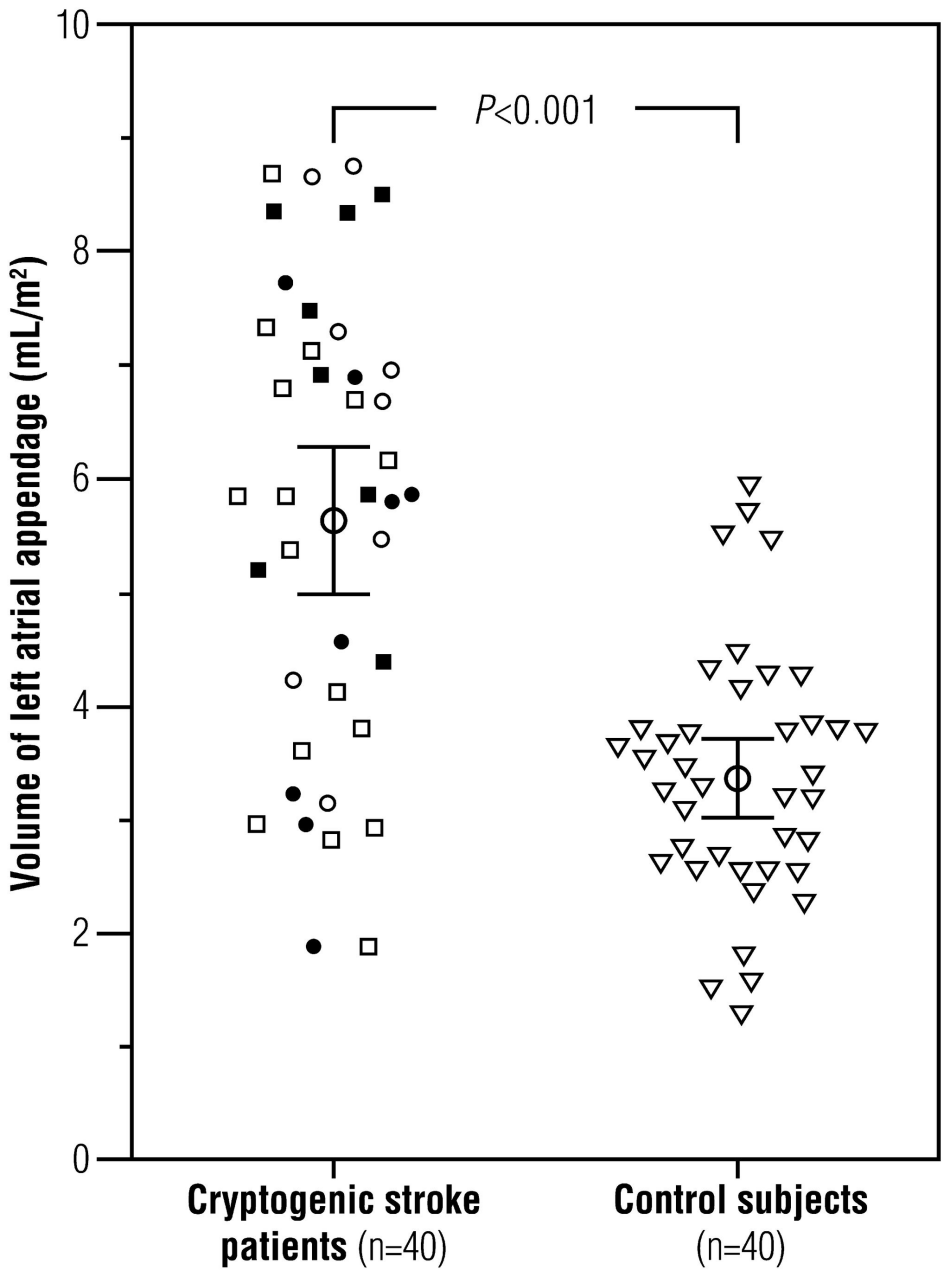
Left atrial appendage (LAA) volumes (mL/m^2^) in 40 cryptogenic stroke/TIA patients (squares/circles) and 40 age- and gender-matched control subjects (triangles), with 95% confidence interval. Mean LAA volume (5.7±2.0 mL/m^2^) was 67% higher in matched patients with stroke/TIA than in control subjects (3.4±1.1 mL/m^2^). Volume enlargement was similar between stroke patients (squares) and TIA patients (circles), and between hypertensive (black) and normotensive (white) patients.

Table 1 shows the clinical characteristics of patients with cryptogenic stroke/TIA and the age- and gender-matched control subjects. Stroke/TIA patients were significantly more obese and had larger BSA. There were no hypertensive patients in the control group whereas 51% of the patients with stroke/TIA suffered from hypertension. Table 2 shows the unadjusted, height-adjusted, and BSA-adjusted LAA and LA volumes between age- and gender-matched pairs. LAA volume was significantly higher in patients with cryptogenic stroke/TIA (5.7±2.0 mL/m^2^) than in control subjects (3.4±1.1 mL/m^2^; *P*<0.001) (Figure 2). LA volume was also significantly higher in stroke/TIA patients (43.9±10.9 mL/m^2^) than in control subjects (32.2±6.7 mL/m^2^; *P*<0.001). Accordingly, LAA was 67% larger and LA 36% larger in patients with cryptogenic stroke/TIA than in control subjects. The differences in LAA and LA volumes between the stroke/TIA patients and control subjects remained highly significant (*P*<0.001) when adjusted for BMI and hypertension, together or separately (Table 2). LAA or LA volumes did not significantly differ between hypertensive (n=16) and normotensive (n=24) nor between obese (n=15) and non-obese (n=25) stroke/TIA patients. 

**Table 1:** Clinical Characteristics of 40 Patients with Acute Cryptogenic Stroke/TIA and 40 Age- and Gender-Matched Control Subjects.

	**Stroke/TIA Patients**	**Control Subjects**	**Significance**
Age, yr	53.9±9.3	53.8±9.0	ns.
Males, n (%)	21 (52.5)	21 (52.5)	ns.
Body mass index, kg/m^2^	28.7±4.8	25.3±4.1	0.002
Body surface area, m^2^	2.0±0.2	1.8±0.2	0.033
Caucasian race, n (%)	40 (100.0)	40 (100.0)	ns.
Hypertension, n (%)	16 (40.0)	0 (0.0)	<0.001
Hyperlipidemia, n (%)	14 (35.0)	16 (40.0)	ns.
Diabetes, n (%)	0 (0.0)	0 (0.0)	ns.
Left ventricle ejection fraction, %	66.0±10.2	63.7±7.7	ns.
Left ventricle dysfunction, n (%)	0 (0.0)	0 (0.0)	ns.
Smokers, n (%)	0 (0.0)	1 (2.5)	ns.

**Table 2:** Left Atrial Appendage (LAA) and Left Atrium (LA) Volume Measurements, Pair-wise Comparison between Patients with Cryptogenic Stroke (n=40) and Age- and Gender-Matched Control Subjects (n=40).

	**Unadjusted**				**Height Adjusted**				**Body Surface Area Adjusted**			
	**Stroke Patients (n=40)**	**Control Subjects (n=40)**	**Sig.**	ANCOVA*	**Stroke Patients (n=40)**	**Control Subjects (n=40)**	**Sig.**	ANCOVA*	**Stroke Patients (n=40)**	**Control Subjects (n=40)**	**Sig.**	ANCOVA*
LAA mean volume	11.1±3.8 mL	6.2±1.9 mL	<0.001	<0.001	6.5±2.2 mL/m	3.7±1.1 mL/m	<0.001	<0.001	5.7±2.0 mL/m^2^	3.4±1.1 mL/m^2^	<0.001	<0.001
LA mean volume	85.5±21.1 mL	59.8±15.3 mL	<0.001	<0.001	50.5±12.6 mL/m	35.1±8.0 mL/m	<0.001	<0.001	43.9±10.9 mL/m^2^	32.2±6.7 mL/m^2^	<0.001	<0.001

* Adjusted for BMI and hypertension.

*Sig.*, Significance.

Based on volume measurements in the control subject population, the upper thresholds for normal LAA volume were 10.1 mL (unadjusted), 5.9 mL/m (height adjusted), and 5.6 mL/m^2^ (BSA adjusted). The upper thresholds for normal LA volume were 90.4 mL, 43.1 mL/m, and 45.6 mL/m^2^, respectively. LAA volume was over 5.6 mL/m^2^ in 23 (58%) of the 40 patients with cryptogenic stroke/TIA, but in only two (5%) of the control subjects (*P*<0.001). LA volume was over 45.6 mL/m^2^ in 20 (50%) patients with cryptogenic stroke/TIA, and in only one (3%) control subject (*P*<0.001).

### LAA and LA Volume in Patients with Cryptogenic Stroke/TIA

Table 3 summarizes the clinical characteristics of all 82 patients with cryptogenic stroke/TIA. Forty-five patients (55%) had an enlarged LAA and 39 (48%) had an enlarged LA using BSA-adjusted threshold values. Both LAA and LA were enlarged in 27 patients, 18 patients had normal LA volume despite enlarged LAA, and 12 patients had enlarged LA volume but normal LAA volume. Cohen’s kappa coefficient indicated minor association (*P*=0.272) between LAA and LA enlargement, and the McNemar test showed this association to be statistically nonsignificant (*P*=0.362). 

**Table 3:** Clinical Characteristics of 82 Patients with Acute Cryptogenic Stroke/TIA.

**Characteristic**	Age, yr	57.8±10.5
	Males, n (%)	57 (69.5)
	Body mass index, kg/m^2^	28.2±4.3
	Body surface area, m^2^	2.0±0.2
	Caucasian race, n (%)	82 (100.0)
	Hypertension, n (%)	42 (51.2)
	Hyperlipidemia, n (%)	27 (32.9)
	Diabetes, n (%)	7 (8.5)
	Smokers, n (%)	21 (25.6)
	Prior stroke, n (%)	11 (13.4)
	Prior myocardial infarction, n (%)	2 (2.4)
**Medication, n (%)**	Aspirin	16 (19.5)
	Warfarin	4 (4.9)
	Clopidogrel	2 (2.4)
	Dipyridamole	2 (2.4)
	Statin	16 (19.5)
Cholesterol, mg/dL*	Low-density lipoprotein	108.3±37.1
	High-density lipoprotein	46.4±19.7

* Data missing for one patient.

### Reproducibility of Volume Measurements

In volume reproducibility measurements we found no significant interobserver difference in LAA (5.0±2.4 mL/m^2^ vs. 5.7±2.5 mL/m^2^, *P*=0.240) or LA volumes (42.2±13.9 mL/m^2^ vs. 44.7±14.5 mL/m^2^, *P*=0.440). Nor did we find significant intraobserver differences in LAA (5.0±2.4 mL/m^2^ vs. 4.7±2.2 mL/m^2^, *P*=0.544) or LA volumes (42.2±13.9 mL/m^2^ vs. 42.1±14.1 mL/m^2^, *P*=0.976). The intra- and interobserver CV% values were 12.6% and 11.2% for LAA, respectively, and 4.6% and 10.0% for LA. The corresponding ICCs were 0.95 and 0.96 for LAA, respectively, and 0.99 and 0.95 for LA.

## Discussion

The main finding of the current study was that more than half (55%) of the consecutive patients with cryptogenic stroke/TIA had an enlarged LAA. In general, LAA volume was 67% larger in patients with cryptogenic stroke/TIA compared to age- and gender-matched control subjects. This result remained unchanged in subgroup analyses with normotensive or non-obese study patients. Interestingly, the observed LAA enlargement was more pronounced than the LA enlargement (67% vs. 36%) and may be more sensitive than LA measurement for detecting a diseased LA-LAA system. A 24-hour Holter has only marginal ability to exclude propensity for PAF [21,22]; therefore, our study patients with stroke/TIA may actually suffer from asymptomatic PAF.

We are not aware of any previous studies investigating LAA volumes in patients with cryptogenic stroke/TIA, or any stroke/TIA patients without AF. It has previously been suggested that measurement of LAA volume could theoretically be valuable for detecting risk patients, because cardioembolic stroke/TIA is a common source of ischemic stroke/TIA, and over 90% of detected LA thrombi are found within the LAA [4,23].LAA enlargement is known to play a part in thrombus formation, independently as well as in association with decreased LAA contraction [10], and there is emerging evidence that specific LAA morphology more frequently indicates thrombus formation [24].

Study subjects were scanned at mid-diastole which provides the most constant image quality in terms of minimal motion artefacts [25]. We found normal LAA mean mid-diastolic volume to be 6.2±1.9 mL (unadjusted), 3.7±1.1 mL/m (height adjusted), and 3.4±1.1 mL/m^2^ (BSA adjusted). In a previous cCT studies by Lacomis et al [26], normal LAA mean volume was 6 mL (range 5–13 mL) in mid-diastole and by Christiaens et al [27], normal LAA mean maximal volume measured in end-systole was 9±3 mL, and normal LAA mean minimal volume measured in end-diastole was 4±2 mL. A study by Nedios et al, reported that normal maximal LAA volume in end-systole was 6.5±1.9 mL [28]. When referred to 143 necropsy LAA casts (4.6 mL) of patients with sinus rhythm, we obtained slightly larger volumes. Necropsy LAA volumes from patients who suffered from FA or cardioembolism were larger compared to our control subjects [29]. Altogether, our results parallel these values, recognizing that the scanning phase in our study was between LAA maximal (end-systole) and minimal (end-diastole) volume. 

We found that there was a narrow range of normal LAA mid-diastolic volumes. Cut-off for normality at the 5.6 mL/m^2^ was exceeded in two control subjects, and in 23 (58%) age- and gender-matched patients with cryptogenic stroke/TIA. This enlargement was not explained by any previously recognized factors causing LA-LAA-system dilatation, such as mitral valve insufficiency or LV dysfunction, because our study groups had no such conditions. The results could also not be explained by arterial hypertension or obesity, as the difference remained the same when the volumes were adjusted for these variants and when the analyses were performed in subgroups without hypertension or obesity. 

Recently, a TEE study utilizing a 3D approach, a MRI study and three CT-based studies reported increased LAA volumes in patients with PAF (13 mL, range 6–51 mL; 8.1±5.1 mL; 10.6±4.1 mL; 8.1±2.5 mL), and even higher LAA volumes in patients with persistent AF (10.5±5.9 mL; 16.5±7.1 mL; 10±5.1 mL) [26,28,30–32]. These findings have been verified by MRI studies in patients with persistent AF and PAF [13,24,32]. Interestingly, our study patients with cryptogenic stroke/TIA had LAA volumes (11.1±3.8 mL) almost as large as the patients with PAF in those studies (13.0±6.1 mL), while the LAA volumes in patients with persistent AF were substantially higher (14.3±6.2 mL; 15.04±07.1 mL; 17.3±6.7 mL). In previous cCT studies by Whisenant et al, both groups suffering from AF with (28.8±13.5 mL) and without (21.7±8.3 mL) stroke/TIA history, had much larger LAA mean volumes compared to our cryptogenic stroke/TIA patients [33,34].

Studies investigating LA volume in patients with cryptogenic stroke/TIA are scarce. LA dilatation has been previously found in only 6.7% of stroke/TIA patients [35], but contradictory results have also been reported [23]. Both studies used LA diameter to determine LA enlargement. For our advantage three-dimensional volume measurements give more accurate results and narrower limits for normality, due to higher measurement accuracy [36,37].

There’s evidence that cardiac embolism constitutes a major mechanism for cryptogenic stroke [7,8,38]. Manina et al [39] found that AF may be found in over 24% of patients assumed to have cryptogenic stroke/TIA using long-term ECG registrations. In another recent study, PAF in episodes from 5–30 seconds was found in 10% of patients with cryptogenic stroke. However, the significance of very short-term (<30 sec) PAF-bursts in patients with cryptogenic stroke is unknown because such arrhythmias have also been found to be common in healthy individuals [40–43]. Our findings raise the question of whether LAA/LA volumetry could improve cardioembolic profile characterization among patients with short-term AF bursts.

The study population included patients with stroke/TIA of undetermined origin or with a clinical suspicion of cardioembolic origin which overemphasizes the finding of enlarged LAA compared to all patients with cryptogenic stroke. On the other hand, the suspicion of cardioembolic stroke/TIA was based on nonspecific symptoms that are common in all stroke/TIA patients, and/or absence of significant carotid disease, which is also frequently encountered in clinical routine. Accordingly, study subjects represent typical patients with cryptogenic stroke. The main study limitation was that number of patients in our study was relatively small and, that our study protocol did not include follow up of the patients to recognize increased risk for atrial arrhythmias, thrombi or recurrent stroke. Despite all efforts and wide information spread among the neurologists, a great amount of stroke/TIA patients were not screened in the study due to heavy clinical work load.

In conclusion, we found that LAA was enlarged in more than half of patients with cryptogenic stroke/TIA. Assessment of LAA and LA volumes may be valuable in the etiological work-up of stroke/TIA without defined etiology. Based on measurements in the control population, mid-diastolic LAA volume larger than 5.6 mL/m^2^ indicates LAA enlargement. Currently, further examination for asymptomatic AF of patients with an enlarged LAA in cCT may be recommended, since AF as an etiology for stroke/TIA may significantly affect treatment of these patients.
